# Beyond the entomological inoculation rate: characterizing multiple blood feeding behavior and *Plasmodium falciparum* multiplicity of infection in *Anopheles* mosquitoes in northern Zambia

**DOI:** 10.1186/s13071-017-1993-z

**Published:** 2017-01-26

**Authors:** Smita Das, Mbanga Muleba, Jennifer C. Stevenson, Julia C. Pringle, Douglas E. Norris

**Affiliations:** 1 The W. Harry Feinstone Department of Molecular Microbiology and Immunology, The Johns Hopkins Malaria Research Institute, Johns Hopkins Bloomberg School of Public Health, 615 North Wolfe Street, Baltimore, MD 21205 USA; 2 Tropical Disease Research Centre, P.O. Box 71769, Ndola, Zambia; 3 Macha Research Trust, P.O. Box 630166, Choma, Zambia

**Keywords:** Malaria, *Anopheles* mosquitoes, Entomological inoculation rate, Multiplicity of infection, Zambia, ICEMR

## Abstract

**Background:**

A commonly used measure of malaria transmission intensity is the entomological inoculation rate (EIR), defined as the product of the human biting rate (HBR) and sporozoite infection rate (SIR). The EIR excludes molecular parameters that may influence vector control and surveillance strategies. The purpose of this study was to investigate *Anopheles* multiple blood feeding behavior (MBF) and *Plasmodium falciparum* multiplicity of infection (MOI) within the mosquito host in Nchelenge District, northern Zambia. Mosquitoes were collected from light traps and pyrethroid spray catch in Nchelenge in the 2013 wet season. All anophelines were tested for blood meal host, *P. falciparum*, and MOI using PCR. Circumsporozoite (CSP) ELISA and microsatellite analysis were performed to detect parasites in the mosquito and MBF, respectively. Statistical analyses used regression models to assess MBF and MOI and exact binomial test for human sex bias. Both MBF and MOI can enhance our understanding of malaria transmission dynamics beyond what is currently understood through conventional EIR estimates alone.

**Results:**

The dominant malaria vectors collected in Nchelenge were *Anopheles funestus* (*sensu stricto*) and *An. gambiae* (*s.s.*) The EIRs of *An. funestus* (*s.s.*) and *An. gambiae* (*s.s.*) were 39.6 infectious bites/person/6 months (ib/p/6mo) and 5.9 ib/p/6mo, respectively, and took multiple human blood meals at high rates, 23.2 and 25.7% respectively. There was no bias in human host sex preference in the blood meals. The SIR was further characterized for parasite genetic diversity. The overall *P. falciparum* MOI was 6.4 in infected vectors, exceeding previously reported average MOIs in humans in Africa.

**Conclusions:**

Both *Anopheles* MBF rates and *P. falciparum* MOI in Nchelenge were among some of the highest reported in sub-Saharan Africa. The results suggest an underestimation of the EIR and large numbers of circulating parasite clones. Together, the results describe important molecular aspects of transmission excluded from the traditional EIR measurement. These elements may provide more sensitive measures with which to assess changes in transmission intensity and risk in vector and parasite surveillance programs.

## Background

Affecting an estimated 214 million people worldwide, malaria is a major public health problem with the burden disproportionately higher in sub-Saharan Africa, where *Anopheles funestus* (*sensu stricto*) and *An. gambiae* (*s.s.*) are the most efficient vectors of *Plasmodium falciparum* malaria [1]. From 2000–2015, there was a 42% decrease in malaria incidence in Africa [1]. Access to ITNs also increased from 2 to 56% from 2000 to 2014, respectively [1]. Such positive progress has been attributed to increased coverage of vector control interventions such as long lasting insecticide-treated nets (LLINs) and indoor residual spraying (IRS), access to rapid diagnostic tests (RDTs), and artemisinin-based combination therapy (ACT) [1]. As vector control activities continue, it will be imperative to conduct surveillance programs that accurately characterize vector foraging behavior and circulating malaria parasites to determine human risk for infection, anopheline behavioral and insecticide resistances, and emergence of parasite drug resistance and increased virulence in endemic populations.

The success of vector control strategies is frequently evaluated pre- and post-intervention by the entomological inoculation rate (EIR) measurement, which is defined as the number of infectious bites per person per time period. It is an indication of malaria transmission intensity by anopheline vectors, and is calculated as the product of the human biting rate (HBR) and the *Plasmodium* species sporozoite infection rate (SIR) [2, 3]. A basic assumption of the HBR component of the EIR is that a mosquito bites (or probes) once and takes a single blood meal per gonotrophic cycle [4, 5]. However, if mosquitoes exhibit multiple blood feeding (MBF) behavior, taking more than one blood meal per gonotrophic cycle, then the HBR increases and subsequently the unadjusted EIR is underestimated [4, 5]. Basic ecological modeling of arthropod disease vectors has demonstrated that an underestimation of the proportion of people bitten may lead to a 2–4-fold increase in the basic reproductive number (R_0_), the number of infected individuals resulting from a single infectious person [6, 7]. Without accounting for MBF, the R_0_ may be underestimated, resulting in an unrecognized and increased risk of malaria within an affected population [4, 5]. Field studies have also illustrated that MBF may be successfully impacted by vector control measures. In southern Zambia, the *An. arabiensis* MBF rate decreased from 18.9% pre-ITN distribution to 9.1% post-distribution, which was attributed to heterogeneity in biting behavior [4, 5]. Understanding the heterogeneity in mosquito feeding behavior may also determine the contribution of different subpopulation of people to pathogen transmission and thus identify risk groups based on sex and age. For example, in western Kenya, young adults were more likely to be bitten by anophelines than older adults and children [8]. Although MBF is not accounted for in the EIR measurement, its estimation is important to accurately define malaria transmission dynamics.

The second component of the EIR calculation, the SIR, is an indicator of vector infectiousness. A metric that is not included in the EIR, but further characterizes infectious sporozoites within the vector is the multiplicity of infection (MOI). The MOI is defined as the number of genetically distinct malaria parasite clones in an infected host [9–11]. Novel parasite genetic diversity arises during meiotic recombination in the mosquito midgut between multiple distinct clones that may originate from a single individual or multiple individuals [12, 13]. Unique clone production has been correlated with the frequency of crossing of parasite clones and subsequent meiotic recombination [12, 13]. Although the implications of MOI on transmission remain understudied, it is possible that MOI shapes not only the efficiency of transmission, but also may contribute to human disease outcomes [14, 15]. Studies in Cameroon have revealed that in comparison to monoclonal infections, multiclonal infections in mosquitoes are found at lower parasitemias, more likely to evade the mosquito immune defenses, and perhaps more efficiently vectored to human hosts [14, 15]. Presence of multiple clones may also lead to competition among clones that have the potential to influence and perhaps enhance parasite transmissibility, parasite genetics, and drive parasite evolution such as drug resistance or increased virulence [13, 14, 16, 17]. The correlation between MOI and clinical severity remains elusive. While some studies have observed a higher MOI in severe cases [18–21], others have not found any such relationship [16, 22–27]. *P. falciparum* MOI may be influenced by anopheline MBF through contact with more than one individual carrying a distinct clone(s) and subsequent meiotic recombination. Conversely, an infected *Anopheles* mosquito exhibiting MBF behavior results in repeat inoculations of genetically diverse malaria parasites to multiple individuals. Furthermore, the *P. falciparum* MOI detected in the human host is often lower and has different distinct alleles when compared to infected anophelines, a finding that has been attributed to gametocytes of clones existing in blood circulation below PCR detection level thresholds and then becoming more abundant and infectious in mosquitoes [13]. Characterizing *Plasmodium* sporozoites within the mosquito, especially in the context of multiple blood feeding behavior, may improve current estimates of MOIs and identify high risk areas or individuals. Both MBF and MOI enhance EIR by describing the extent, efficiency, and dynamics of malaria transmission. Together, they can serve as potentially important tools for surveillance in malaria endemic areas.

Located along Lake Mweru in northern Zambia and bordering the Democratic Republic of Congo (DRC), Nchelenge District experiences intense malaria transmission year-round [28]. Both LLIN distribution and IRS campaigns have been implemented in Nchelenge since 2006, but the area remains at high risk for malaria [29]. The major vectors of *P. falciparum* transmission in this area are *An. funestus* (*s.s.*) and *An. gambiae* (*s.s.*), both of which are highly anthropophilic [28, 30]. *An. funestus* (*s.s.*) is the dominant vector with a higher EIR year-round compared to *An. gambiae* (*s.s.*), which increases and then declines dramatically from the wet to dry seasons [30]. Recent findings indicated that *An. funestus* (*s.s.*) is the predominant vector in streamside areas, whereas *An. gambiae* (*s.s.*) is most abundant in lakeside areas during the wet season [30]. The aims of this study were to further characterize the fundamental components of the EIR measurement by determining the multiple blood feeding frequency and human host sex preference of *An. funestus* (*s.s.*) and *An. gambiae* (*s.s.*), and the *P. falciparum* MOI in infected mosquitoes.

## Methods

### Study area

**Fig. 1:**
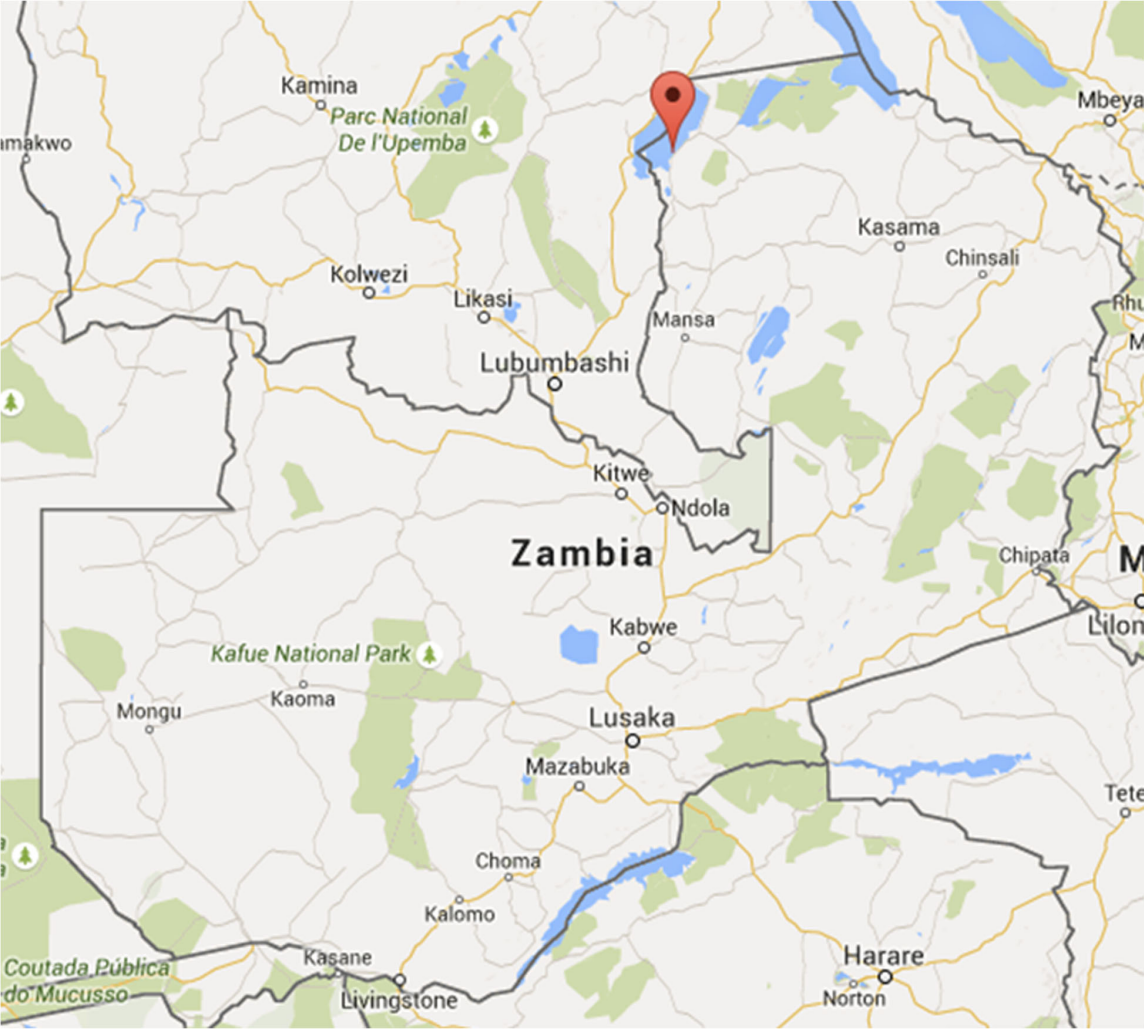
Nchelenge District field site is in northern Zambia and represents an area with high malaria transmission

**Fig. 2:**
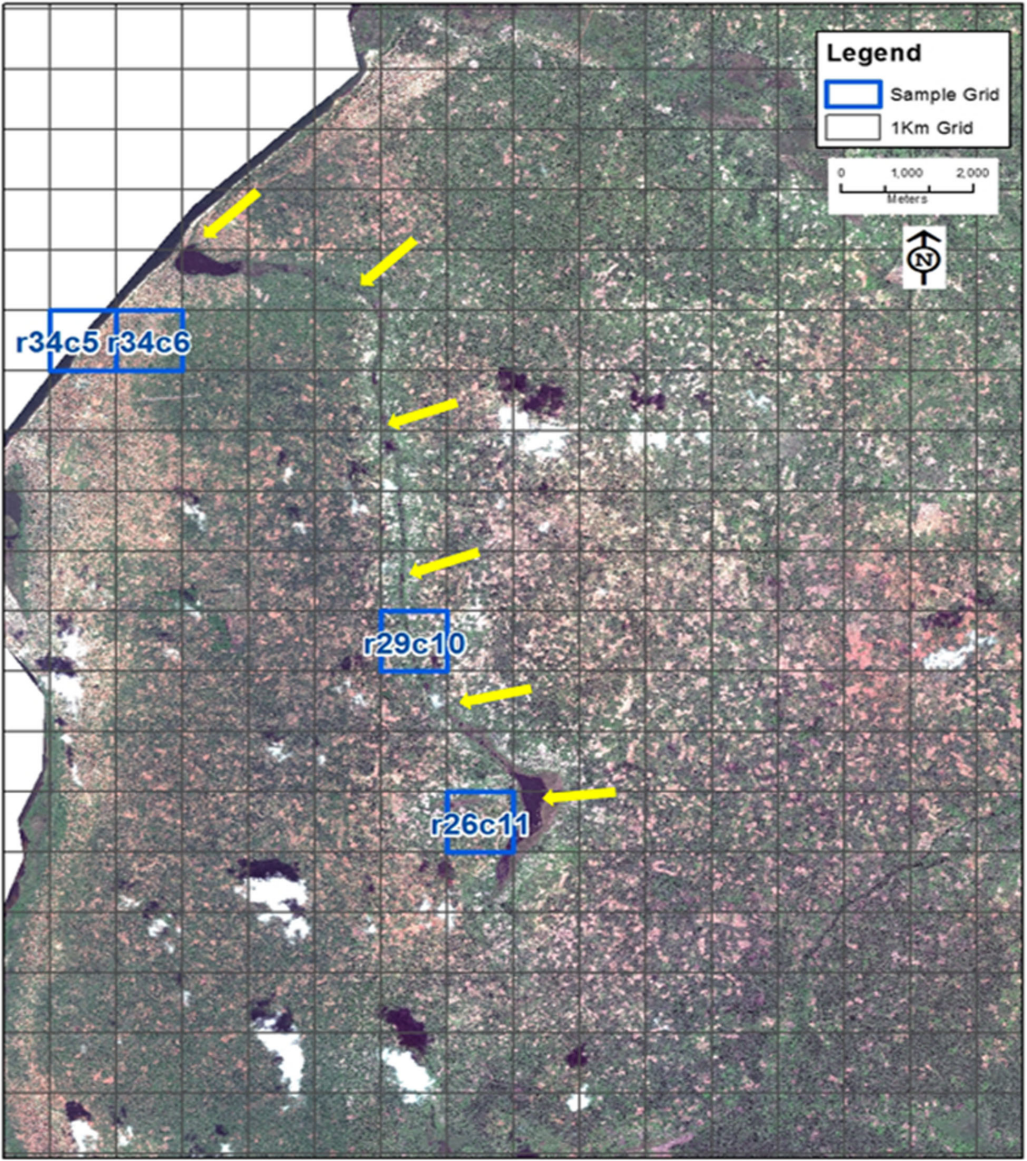
Satellite image of the study area in Nchelenge District. The 1-km^2^ grids for study collections are highlighted in blue. Katuna, Yenga, and Malulu villages are located in grids r34c5 and r34c6, Kapande B village and Mutepuka is located in grids r29c10 and r26c11, respectively. The white area on the left side of the image represents Lake Mweru. The yellow arrows point to Kenani Stream that flows into Lake Mweru

This study was conducted in collaboration with the Johns Hopkins Southern Africa International Centers for Excellence in Malaria Research (ICEMR) project in Nchelenge District, Luapula Province, in northern Zambia (9°19.115'S, 28°45.070'E; Fig. 1). Mean elevation is approximately 807 m above sea level and habitat is of a marsh ecotype. Nchelenge lies along the eastern perimeter of Lake Mweru, which serves as a border between southeastern Democratic Republic of Congo (DRC) and northern Zambia. Kenani Stream is a major water body that flows from south to north through the study area in Nchelenge and into Lake Mweru (Fig. 2). The region experiences three seasons: a single rainy season from November to May, a cool dry season from May to August, and a hot dry season from August-November. Malaria prevalence in this region is high, 38% by microscopy and 56% by RDT in children under the age of 5, and is considered holoendemic [29, 31]. Mosquito sampling was performed at households enrolled in the ICEMR program located within two defined 1-km^2^ grids along both Lake Mweru and another two 1-km^2^ grids inland near Kenani Stream (Fig. 2). These villages are representative of the local demography and landscape, and frequent seasonal movement of local people from fishing to farming endeavors.

### Mosquito collection and handling

From March 5-April 25, 2013 (rainy season), mosquitoes were collected by Center for Disease Control light traps (CDC LTs) and pyrethroid spray catches (PSCs) in five villages: three lakeside villages in grids r34c5 and r34c6 (Katuna, Yenga, and Malulu) and two streamside villages in grids r26c11 and r29c10 (Mutepuka and Kapande B) (Fig. 2). These same rainy season study villages and their respective mosquito collection and handling have been previously characterized [30]. CDC LT and PSC collections were conducted as previously described [28]. Briefly, lakeside and streamside villages were intensely sampled on alternate days of each week. Sampling took place at 39 lakeside and 38 streamside households, totaling 77 CDC LT and 77 PSC collections. Traps were hung indoors next to sleeping persons under LLINs, approximately 1.5 m above the floor from 18:00 to 6:00 h. PSCs were performed in the morning between 6:00 and 10:00 h in selected households using a 100% synthetic aerosol pyrethroid applied to ceilings, eaves, and walls [30].

All field-caught mosquitoes were killed immediately by freezing. Female anophelines were separated and morphologically identified to species using a dissecting microscope and dichotomous key [32, 33]. Mosquitoes were stored on desiccant either at room temperature or frozen at -20 °C until laboratory processing, which took place at Johns Hopkins Bloomberg School of Public Health (JHBSPH) in Baltimore, Maryland.

### Mosquito DNA preparation and PCR

The head and thorax of each collected anopheline were separated from the abdomen and a modified salt extraction method was used to extract DNA from the abdomen [34]. Morphological identifications were confirmed using a polymerase chain reaction (PCR) specific for members of the *An. funestus* or *An. gambiae* complexes [35, 36]. If neither assay could confirm species identity, then a PCR targeting the ITS2 gene that amplifies a range of African anopheline species-specific fragments was used [35].

All specimens, regardless of visually-determined fed or unfed status, were tested for blood meal host using the Kent et al. (2005) multiplex PCR, which targets the mitochondrial cytochrome b gene producing a range of mammalian host specific bands [34]. A modification to the Kent PCR enhanced detection of human hosts. The multiplexed method amplifying the human cytochrome b gene remained the same, except that a new set of human primers replaced the original human forward primer. The new forward primer was FOR16068: 5'-GAC TCA CCC ATC AAC AAC CG-3' and the reverse primer was REV16260: 5'-GGC TTT GGA GTT GCA GTT GA-3' to produce a 193 base pair (bp) amplicon [28, 30]. The more sensitive Fornadel et al. (2010) PCR, which amplifies a 98 bp region of the mammalian cytochrome b gene followed by a restriction fragment length polymorphism (RFLP) assay specific to host of interest, was performed on samples that did not amplify a band(s) for blood meal host by the modified Kent et al. PCR [37]. If mosquitoes were morphologically classified as “unfed”, but contained a blood meal either PCR method, then those mosquitoes were labeled as “unfed but fed” [28].

DNA from mosquito abdomens was also used to test for the presence of the *P. falciparum* parasite using a PCR described by Fornadel et al. (2010) that amplifies a small 183 bp portion of the *P. falciparum* cytochrome b gene and has been shown to be more sensitive and reliable than other commonly used PCR-based assays [38].

### Circumsporozoite enzyme-linked immunosorbent assay (CSP-ELISA) for *Plasmodium falciparum* detection in anophelines

The CSP-ELISA method adapted from Burkot et al. [39] and the Malaria Research and Reference Reagent Resource Center (MR4) was used to specifically detect *P. falciparum* circumsporozoite protein (CSP) in the mosquito head and thorax. Due to the large number of anophelines, a subsample of mosquitoes was selected for CSP-ELISA: 695/2417 (28.8%) *An. funestus* (*s.s.*) and 521/564 (92.4%) *An. gambiae* (*s.s.*) The minimum sample sizes for *An. funestus* (*s.s.*) and *An. gambiae* (*s.s.*) were calculated based on a 90% confidence interval using a hypothesized SIR of 0.02 and specified error of ± 0.01, which was *n* = 421. The hypothesized SIR for the vectors was based on previous preliminary 2012 wet and dry season collections. The specimens were randomized across all collection dates using Microsoft Excel (2011). Mosquito samples that had absorbance values greater than two-fold the negative control absorbance were considered CSP positive.

### Human microsatellite analysis in anophelines

Multiple blood feeding behavior was determined by amplifying and sequencing human microsatellites in human-fed anophelines. A subsample of human-blooded *An. funestus* (*s.s.*) (*n* = 245)*,* and *An. gambiae* (*s.s.*) (*n* = 140) were used for microsatellite analysis. Previous studies in Africa [8, 40] have recorded multiple blood feeding rates in both *An. funestus* (*s.s.*) and *An. gambiae* (*s.s.*) to range from approximately 10–20%. The sample size was calculated based on a 95% confidence interval, an assumed multiple blood feeding rate of 0.15 with a specified error of ± 0.05, which was *n* = 196. Due to the small number of total fed *An. gambiae* (*s.s.*) in the collection, the minimum sample size for this species was not attained. The specimens were randomized across all collection dates using Microsoft Excel (2011). Four loci were used to determine human allelic diversity and estimate the minimum number of contributors to a blood meal as previously described [4]. Primers fluorescently labeled with HEX and FAM were used to amplify the CSF1PO, THO1, Penta D STR (Penta D), and Silver-STR (D13S317) loci [41]. Minor primer modifications by Jiang et al. [42] were made to all primers (Table 1). The 20 μl PCR reaction for each microsatellite contained 10 mM Tris, pH 8.3, 50 mM KCl, 1.5 mM MgCl_2_, 0.01% gelatin, 200 μM dNTPs, 2.0 U *Taq* polymerase, 20 pmol each forward and reverse primers, and 2 μl template DNA.

**Table 1:** Microsatellites and their corresponding label and primer sequences for multiple blood feeding assay and gender preference from engorged *Anopheles* mosquitoes

Primer	Primer sequence (5’ to 3’)
CSF1PO A	/HEX/ACTCCAGGGCAGTGTTCCA
CSF1PO B	AGCCCATTCTCCAGCCTCC
D13S317 A	/HEX/CATGGTATCACAGAAGTCT
D13S317 B	CCAAAAAGACAGACAGAAAGATAG
PentaD A	/HEX/AAGTAGGATCACTTGAGCCTG
PentaD B	CAAGTCCTTTTTTAGATATGTGA
THO1 A	/6-FAM/ATTCAAAGGGTATCTGGGCTCTG
THO1 B	TGGGCTGAAAAGCTCCCGATTAT
Amelogenin A	/6-FAM/CCCTGGGCTCTGTAAAGAATAGT
Amelogenin B	ATCAGAGCTTAAACTGGGAAGCTG

To determine the sex of the human host in fed mosquitoes, the Amelogenin locus was amplified using 6-FAM-labeled primers as previously described (Table 1) [4]. The 50 μl PCR reaction for Amelogenin contained 10 mM Tris, pH 8.3, 50 mM KCl, 1.5 mM MgCl_2_, 0.01% gelatin, 400 μM dNTPs, 2.0 U *Taq* polymerase, 25 pmol each forward and reverse primers, and 2 μl template DNA.

One microliter each from CSF1PO, THO1, Penta D, and D13S317 PCR reactions and 2 μl Amelogenin PCR reaction were multiplexed together with 15 μl formamide and 0.5 μl GeneScan-500 Rox size standard (Applied Biosystems Inc., Foster City, California) and incubated for 5 min at 95 °C. The samples were then prepared for shipment and subsequent fragment analysis to the DNA Analysis Facility on Science Hill (Yale University, New Haven, Connecticut). Sequencing results were analyzed using Peak Scanner 2 DNA fragment analysis software (Applied Biosystems Inc., Foster City, California) at the Johns Hopkins Bloomberg School of Public Health (JHBSPH). The software separated, profiled, and calculated sizes of DNA fragments based on the sequencing data. A blood meal was considered to be from multiple human hosts if there were three or more alleles at any microsatellite locus.

### *Plasmodium falciparum* multiplicity of infection (MOI)

Anophelines that were positive for *P. falciparum* infection by the *cytb* PCR or CSP-ELISA were genotyped to determine the parasite MOI. The repetitive regions block 2 and 3 of merozoite surface protein 1 and 2 (*msp-1* and *msp-2*), respectively, were amplified by a nested PCR, and the RII repeat region of glutamate receptor protein (*glurp)* of *P. falciparum* was amplified by a semi-nested PCR [16]. The second nested reaction in the overall PCR protocol detects allelic variants including K1, MAD20, and RO33 families of *msp-1* block 2, the FC27 and 3D7/IC families of *msp-2* block 3, and the RII (GLURP) block of *glurp* [16]. Following gel electrophoresis, amplicon sizes were characterized using FluorChem Image Analyzer (Protein Simple) to determine distinct alleles. For each isolate, the *msp-1, msp-2,* and *glurp* allelic families were described. If a single PCR amplicon was detected at only one locus (*msp1*, *msp2*, or *glurp*), the parasite was considered to be monoclonal. If more than one PCR amplicon was detected at any locus, then the infection was considered to contain multiple *P. falciparum* genotypes. The number of bands for *msp-1* and *msp-2* were determined by adding the bands observed for K1, MAD20, RO33 families, and the FC27 and 3D7/IC families, respectively [16, 43]. The largest number of bands at any of the loci (*msp-1*, *msp-2*, *glurp*) was considered the overall MOI in the infected mosquito vector [16, 43]. The alleles for each family were placed into bins with a 40 bp width to determine the number of distinct alleles [44–46]. The mean MOI was calculated by dividing the sum of each sample’s overall MOI by the number of positive samples.

### Statistical analysis

A logistic regression model with random effects to account for repeated sampling at two geographically distinct areas, Lake Mweru and Kenani Stream (lake versus stream), and clustering of traps was used to compare *An. funestus* (*s.s.*) and *An. gambiae* (*s.s.*) multiple blood feeding behavior and overall detection rates of parasite clones among loci and allelic families in anopheline vectors. Bias in sex preference of anopheline blood meals was identified by exact binomial test where the hypothesized probability of males was 0.50. A negative binomial regression with random effects for repeated collections between lake and stream, and clustering of traps was used to investigate the overall *P. falciparum* MOI among antigenic markers, as well as between and within infected *An. funestus* (*s.s.*) and *An. gambiae* (*s.s.*). Statistical significance was defined as a *P*-value less than or equal to 0.05. All statistical analyses were performed using STATA version 11 software.

## Results

### Species identification

Two thousand nine hundred and eighty-nine *Anopheles* mosquitoes were caught from 77 households, most of which were *An. funestus* (*s.s.*) (80.9%, *n* = 2,417), followed by *An. gambiae* (*s.s.*) (18.9%, *n* = 564) and *An. leesoni* (0.2%, *n* = 8). *An. leesoni* was not included in this study due to its small sample size. As described previously [30], of the 2,981 *An. funestus* (*s.s.*) and *An. gambiae* (*s.s.*), 2,024 (67.9%) and 957 (32.1%) were collected by CDC LTs and PSCs, respectively. The lakeside collections (*n* = 134) showed smaller numbers of malaria vectors collected by PSCs (11.9%, *n* = 16) compared to CDC LTs (88.1%, *n* = 118). Similarly, PSCs collected fewer anophelines compared to CDC LTs in the streamside collections (*n* = 2,847), 941 (33.1%) and 1,906 (66.9%), respectively.

### Blood feeding behavior

All *Anopheles* mosquitoes regardless of morphological abdominal status were tested for blood meal host due to the possibility that there may be mosquitoes that appear “unfed”, but contain blood (“unfed but fed”) as determined by molecular assays [28]. A total of 18.4% (444/2417) *An. funestus* (*s.s.*) and 17.7% (100/564) *An. gambiae* (*s.s.*) were visually fed, of which 430 (96.8%) and 90 (90%) were human-fed, respectively. There were 24 *An. funestus* (*s.s.*) and 6 *An. gambiae* (*s.s.*) that had mixed human and goat blood meals. Of the anophelines that were morphologically classified as “unfed”, 415 were positive for a human host: 344 *An. funestus* (*s.s.*)*,* 68 *An. gambiae* (*s.s.*), and 3 *An. leesoni*. Mixed human and goat blood meals were detected in 7 *An. funestus* (*s.s.*) and 3 *An. gambiae* (*s.s.*). The resulting average human blood indices (HBIs), defined as the proportion of human blood meals, for *An. funestus* (*s.s.*) and *An. gambiae* (*s.s.*) were 0.96 and 0.95, respectively.

### *Plasmodium falciparum* detection

By CSP-ELISA, overall *P. falciparum* positivity rates using both CDC LT and PSC collections were 2.7% (19/695) for *An. funestus* (*s.s.*) and 3.1% (16/521) for *An. gambiae* (*s.s.*). By PCR, 1% (24/2434) of *An. funestus* (*s.s.*) and 1.8% of *An. gambiae* (*s.s.*) (10/564) were *P. falciparum* positive. Of the malaria vectors that were tested by both CSP-ELISA and PCR, 1% (7/695) of *An. funestus* (*s.s.*) and 0% (0/521) of *An. gambiae* (*s.s.*) were malaria-positive.

### Multiple blood feeding

Of the 385 human-fed anophelines tested for MBF by human microsatellite analysis, 280 (72.7%) were successfully genotyped at more than one locus. This subset was composed of 179 *An. funestus* (*s.s.*) and 101 *An. gambiae* (*s.s.*). Mosquitoes determined to be “fed” by the Fornadel et al. PCR made up 75.2% of the failed microsatellite samples (79/105), suggesting that the quantity of host DNA may have been too limited or highly degraded for the microsatellite assay. Of the four loci, CSP1PO had the lowest failure rate (40.3%, 155/385) and THO1 had the highest failure rate (57.4%, 221/385). In total, 27.5% (106/385) of genotyped anophelines failed at all loci. The overall multiple blood feeding frequencies for *An. funestus* (*s.s.*) and *An. gambiae* (*s.s.*) were 24% (43/179) and 27.7% (28/101), respectively, and no evidence of a difference was found between these rates (OR = 1.1, 95% CI: -0.62–1.8, *P* = 0.83).

Of the 280 human-fed anophelines detected by microsatellite analysis, 97 were “unfed but fed”: 55 *An. funestus* (*s.s.*) and 42 *An. gambiae* (*s.s.*). The MBF rate was 16.4% (9/55) and 40.5% (17/42) for *An. funestus* (*s.s.*) and *An. gambiae* (*s.s.*), respectively.

### Human sex preference

The sex of detected human hosts in fed *Anopheles* mosquitoes was successfully amplified at the Amelogenin locus in 80.5% samples (310/385). The proportion of human blood meals detected in both *Anopheles* vectors belonging to males was not significantly different to that of females: 53.2% (*n* = 165) for males and 46.8% (*n* = 145) for females (*P* = 0.20). When compared to the Nchelenge District-wide ratio of human males to females as determined by the Zambia 2010 census [47], 48.5 to 51.5%, the mosquito feeding remained unbiased (*P* = 0.20).

### Multiplicity of infection (MOI)

Both *P. falciparum* PCR and ELISA positive anophelines (*n* = 62) were used to investigate the MOI. The overall rate of successful amplification was 80.6% (50/62). At the *msp-1*, *msp-2*, and *glurp* loci in 50 *P. falciparum* infected mosquitoes 86% (*n* = 43), 94% (*n* = 47), and 48% (*n* = 24) were successfully amplified (Table 2). In the successfully amplified samples, multiple and single clones were detected in 47 (94%) and 3 (6%) infected mosquitoes, respectively. The mean *P. falciparum* MOIs for successfully amplified loci were 5.3 (Standard Deviation, SD = 3.7), 5.8 (SD = 3.8), and 1.6 (SD = 1.0) for *msp-1*, *msp-2*, and *glurp,* respectively, and the overall mean MOI was 6.4 (SD = 4.1) (Table 3). The MOIs of *msp-1* and *msp-2* were 3.5 and 3.8 times greater that of *glurp* (*msp-1*: RR = 3.3, 95% CI: 2.3–5.1, *P* < 0.001; *msp-2*: RR = 3.78, 95% CI: 2.6–5.6, *P* < 0.001), and no evidence of a difference between the *msp-1* and *msp-2* MOIs (RR = 1.1, 95% CI: 0.87–1.38, *P* = 0.44). The odds of detecting *P. falciparum* clones at *msp-1* and *msp-2* loci were 6.9 and 11 times greater, respectively, compared to *glurp* (*msp-1*: OR = 6.9, 95% CI: 2.56–18.8, *P* < 0.001; *msp-2*: OR = 11, 95% CI: 3.83–31.6, *P* < 0.001). The odds of detecting parasite clones between *msp-1* and *msp-2* (OR = 1.7, 95% CI: 0.61–4.7, *P* = 0.31) did not support a difference.

**Table 2:** Summary of *msp-1*, *msp-2*, and *glurp* positive detection rates in successfully amplified *P. falciparum*-infected *Anopheles* mosquitoes (*n* = 50) from March-April 2013 in Nchelenge District, northern Zambia

Gene	No. positive (%)
*msp-1*	43 (86)
K1	40 (80)
MAD20	24 (48)
RO33	26 (52)
*msp-2*	47 (94)
FC27	44 (88)
IC/3D7	38 (76)
*glurp*	24 (48)

**Table 3:** Overview of *P. falciparum* infection complexity in successfully amplified *Anopheles* mosquitoes (*n* = 50) from March-April 2013 in Nchelenge District, northern Zambia

Gene	Mean MOI ± SD	No. of distinct genotypes
*msp-1*	5.3 ± 3.7	31
K1	2.2 ± 1.5	11
MAD20	2.8 ± 1.8	8
RO33	2.7 ± 1.8	12
*msp-2*	5.8 ± 3.8	37
FC27	3.7 ± 2.5	21
IC/3D7	2.3 ± 1.3	16
*glurp*	1.6 ± 1.0	12
Overall MOI	6.4 ± 4.1	

*Abbreviation*: *MOI* multiplicity of infection; *SD* standard deviation

**Fig. 3:**
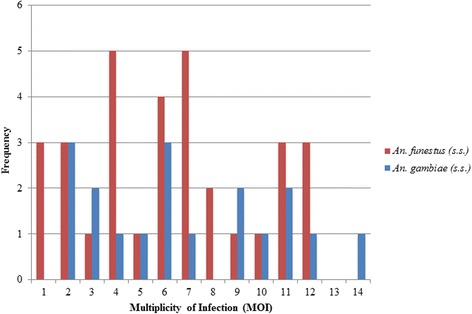
Frequency distribution of *P. falciparum* multiplicity of infection (MOI) in infected *Anopheles funestus* (*s.s.*) and *An. gambiae* (*s.s.*) in Nchelenge District

The successfully genotyped infected mosquitoes comprised of 34 (68%) *An. funestus* (*s.s.*) and 16 (32%) *An. gambiae* (*s.s.*). The ranges in numbers of parasite clones for *An. funestus* (*s.s.*) and *An. gambiae* (*s.s.*) were similar: 1–12 and 1–14 clones, respectively (Fig. 3). The mean MOIs of *An. funestus* (*s.s.*) and *An. gambiae* (*s.s.*) were comparable, 5.9 and 7.5, respectively (RR = 1.2, 95% CI: 0.70–2.13, *P* = 0.48). There was no evidence of a difference in MOI between *An. funestus* (*s.s.*) and *An. gambiae* (*s.s.*) at *msp-1, msp-2,* and *glurp* (*msp-1*: RR = 0.86, 95% CI: 0.44–1.67, *P* = 0.66; *msp-2:* RR = 1.53, 95% CI: 0.84–2.80, *P* = 0.16; *glurp*: RR = 0.84, 95% CI: 0.35–2.02, *P* = 0.70). There was also no evidence of differences in the detection success of *msp-1*, *msp-2*, and *glurp* between both *Anopheles* mosquitoes (*msp-1*: OR = 0.56, 95% CI: 0.09–3.49, *P* = 0.53; *msp-2*: OR = 1.33, 95% CI: 0.25–7.04, *P* = 0.74; *glurp*: OR = 0.74, 95% CI: 0.21–2.53, *P* = 0.63). The MOIs of the three loci were also compared within vector species. For *An. funestus* (*s.s.*), the MOIs of *msp-1* and *msp-2* were both approximately 3.6 times higher than *glurp* (*msp-1*: RR = 3.58, 95% CI: 2.29–5.62, *P* < 0.001; *msp-2*: RR = 3.62, 95% CI: 2.31–5.68, *P* < 0.001). In *An. funestus* (*s.s.*)*,* the MOI of *msp-1* compared to *msp-2* loci was identical (RR = 1, 95% CI: 0.8–1.3, *P* = 0.96) and the detection success of the three loci showed that *msp-1* and *msp-2* amplified better than *glurp* (*msp-1*: OR = 16.5, 95% CI: 4.0–68.5, *P* < 0.001; *msp-2*: OR = 9.1, 95% CI: 2.46–33.6, *P* = 0.001). The odds of amplification between *msp-1* and *msp-2* did not suggest a difference (OR = 0.52, 95% CI: 0.14–1.95, *P* = 0.33). For *An. gambiae* (*s.s.*)*,* the *msp-1* and *msp-2* MOIs were 3.5 and 4.3 times greater, respectively, compared to *glurp* (*msp-1*: RR = 3.47, 95% CI: 1.75–6.87, *P* < 0.001; *msp-2*: RR = 4.26, 95% CI: 2.19–8.29, *P* < 0.001), and *msp-2* MOI was 1.2 times that of *msp-1* (RR = 1.25, 95% CI: 0.86–1.82, *P* = 0.25). The odds of detecting *msp-1* compared to *glurp* and *msp-2* to *msp-1* in *An. gambiae* (*s.s.*) were greater, but not significant (*msp-1*: OR = 6.74, 95% CI: 0.92–49.1, *P* = 0.06; *msp-2*: OR = 8.02, 95% CI: 0.72–89.2, *P* = 0.09). *Msp-2* had greater odds of detection compared to *glurp* (OR = 29.4, 95% CI: 2.92–296.2, *P* = 0.004).

For each *P. falciparum* gene locus with more than one allelic family, successfully genotyped parasites in anophelines were characterized (Table 2). For the *msp-1* locus, the detection odds of MAD20 and RO33 families were lower compared to K1 (MAD20: OR = 0.22, 95% CI: 0.09–0.55, *P* = 0.001; RO33: OR = 0.27 95% CI: 0.11–0.66, *P* = 0.004). The odds of detection of RO33 compared to MAD20 (OR = 1.2, 95% CI: 0.53–2.60, *P* = 0.69) supported no difference between markers. The odds of malaria parasite detection in infected mosquitoes at *msp-2* FC27 and IC/3D7 families suggested no evidence of a difference (OR = 1.96, 95% CI: 0.76–5.05, *P* = 0.16).

Within each allelic family of the *msp-1* (K1, MAD20, RO33), *msp-2* (FC27, IC/3D7), and *glurp* (GLURP) genes, distinct alleles were enumerated. At the *msp-1* and *msp-2* loci, 31 and 37 distinct alleles were observed, respectively (Table 3). For *glurp*, 12 unique alleles were identified (Table 3). K1, RO33, and FC27 showed greater parasite diversity (Table 3), suggesting that parasite clones containing polymorphisms in these allelic families were predominant.

## Discussion

Despite their influence on malaria epidemiology, anopheline MBF and *P. falciparum* MOI are traditionally not considered when assessing the EIR. Here, these parameters were evaluated in parallel with HBR and SIR to more accurately investigate the foraging behavior of malaria vectors in Nchelenge District. Collections of mosquitoes in this district revealed *An. funestus* (*s.s.*) and *An. gambiae* (*s.s.*) as the main malaria vectors both of which were highly anthropophilic. As reported previously from the same 2013 collection presented in this study, a large proportion of total anophelines in this collection, especially *An. funestus* (*s.s.*), were trapped in the streamside households compared to the lakeside households, illustrating heterogeneity in malaria vector species [30]. This may be attributable in part to differences in breeding sites, vegetation for resting, human population density, and application of vector control, among others, between the lake and stream areas. The trapping methods used in this study, CDC LT and PSC, target indoor mosquitoes only, but the possibility remains that these same vectors are also feeding intensely on humans or other mammals and additionally resting outdoors. As a result, it will be important to perform additional further studies that include outdoor foraging and resting collections.

*An. funestus* (*s.s.*) and *An. gambiae* (*s.s.*) exhibited MBF behavior at rates of 24% and 27.7%. Observed differences in MBF frequencies between vectors were not observed, suggesting that *An. funestus* (*s.s.*) and *An. gambiae* (*s.s.*) feed on multiple people in a single gonotrophic cycle at similar rates. These rates are much higher than the 11–14% multiple blood feeding rates reported for *An. funestus* (*s.s.*) and *An. gambiae* (*s.s.*) in western Kenya [8], 10% rate for *An. gambiae* (*s.s.*) in Nigeria [44], 9% rate for *An. funestus* (*s.l.*) in Tanzania [40], and even the high rate of 18.9% reported for *An. arabiensis* prior to the introduction of LLINs in southern Zambia [4]. Multiple blood meals were detected in morphologically unfed mosquitoes that were PCR confirmed as fed, suggesting that the microsatellite assay is highly sensitive in identifying multiple meals even in incomplete or partially digested blood meals. Furthermore, our findings on multiple blood feeding behavior in these *Anopheles* species reveal that the process of taking a blood meal is disrupted more frequently than our previous estimates indicated [28]. Factors that may influence multiple blood feeding include host defensive behavior, response to vector control, and parasite modulation of vector feeding behavior [4, 5, 8, 40, 48–58].

The epidemiologic impact of multiple blood feeding behavior is that it increases the human biting rate, leading to an increase in the reproductive number R_0_. For example, when a 20% daily multiple blood feeding rate is accounted for in the vectorial capacity equation, the predicted result is a 44% increase in the number of new infectious bites [59, 60]. This highlights the sensitivity of vectorial capacity to even small changes in the HBR. A simpler, more practical parameter that directly corresponds to malaria risk is the EIR, defined as the number of infective bites per individual per time period. In Nchelenge District, EIRs during the 2013 wet season for *An. funestus* (*s.s.*) and *An. gambiae* (*s.s.*) were 39.6 ib/p/6mo and 5.9 ib/p/6mo, respectively [30]. If multiple blood feeding is considered, the resulting EIRs would increase to 48.8 ib/p/6mo for *An. funestus* (*s.s.*) and 7.4 ib/p/6mo for *An. gambiae* (*s.s.*). An increased biting rate decreases the vector population size needed to sustain malaria parasite transmission and smaller populations may be more difficult to control and eliminate [61]. Accordingly, vector control programs that aim to reduce human infection by decreasing the *Anopheles* population may have to be more efficient for the same impact if a single mosquito could contribute to multiple human infections during a single gonotrophic cycle.

The identification of human male or female blood in fed *Anopheles* vectors can reveal heterogeneities in risk and potentially target control strategies. In Nchelenge District, there was no bias in sex preference for blood feeding by *An. funestus* (*s.s.*) and *An. gambiae* (*s.s.*) considering the proportion or males to females according to district census data, suggesting that males and females are bitten, equally, and both contribute and are exposed to local transmission. Similar research in southern Zambia also found no significant difference in the biting preference of *An. arabiensis* [5], whereas other studies of *An. funestus* (*s.s.*) and *An. gambiae* (*s.s.*) in Kenya and Tanzania observed a feeding bias towards young children and males, respectively [40, 46]. DNA profiling of household inhabitants would have provided additional details about the human age groups of blood meals [40]. Further studies are warranted to better understand sex or age preference of mosquitoes, and how human behavior, ITN use, and human malaria epidemiology may affect blood feeding preferences.

The second component of the EIR, the SIR, does not traditionally include a characterization of the parasite genetic diversity found within an infected vector and human host, and its implications for transmission in a malaria endemic population. Although *P. falciparum* MOI does not affect the EIR *per se*, it does describe an important aspect of vector- host transmission that is not included in the calculation. To the authors’ knowledge, there have been no other reports of *P. falciparum* MOI in field-caught mosquitoes. Collections in this study revealed multiple clones in over 90% of infected anophelines ranging from 1–12 and 1–14 clones in *An. funestus* (*s.s.*) and *An. gambiae* (*s.s.*), respectively. The overall MOI was 6.4, indicative of a high transmission setting [11, 43, 62], and is among some of the highest MOIs reported when compared to that of infected humans in Africa: 3.7 in Tanzania, 3.4 in Cote d’Ivoire, 3.2 in Mauritania, 3.0 in Uganda, 2.0 in western Kenya, 1.9 in eastern Sudan, and 1.5 in Nigeria and the Gambia [13, 16, 26, 43, 63–66]. The overall MOI was comparable between the two vector species, with 5.9 and 7.5 clones in *An. funestus* (*s.s.*) and *An. gambiae* (*s.s.*), respectively, and the numbers and frequency of clones harbored by both vectors were also similar. Furthermore, it is interesting that, although the proportion of *An. gambiae* (*s.s.*) is smaller within the *P. falciparum*-positive anopheline population, this vector has a higher average MOI than that of *An. funestus* (*s.s.*). This may be due to the small sample size and warrants additional studies to confirm and understand interspecies variation in MOI. Eighty unique *P. falciparum* alleles were identified in the vectors and were most diverse at the *msp-1* and *msp-2* loci, with the K1 and FC27 alleles being predominant, respectively. The successful PCR amplification of *msp-1* and *msp-2* compared to *glurp* has been reported previously and suggests the importance of including multiple loci to better estimate the MOI [16, 67–69]. The study results indicate that *glurp* may simply not be as polymorphic as the *msp* loci or is weakly amplified, and therefore is a poor marker for genetic diversity [16].

The number of unique clones within an infected anopheline is indicative of transmission success, highlighting the crucial role the mosquito has in sustaining parasite diversity [15]. In the context of *Anopheles* MBF behavior, *P. falciparum* genetic diversity may be positively influenced by the acquisition and transmission of multiple distinct clones by feeding on several individuals in a single gonotrophic cycle. Further studies will need to be performed to better define the potential interaction between foraging behavior and parasite population structure, especially in the context of parasite evolution and emergence of drug resistance or increased virulence. Preliminary analysis of MOI in 25 infected human DBS collected in Nchelenge as part of a separate ICEMR survey in April 2013 showed a much smaller range of multiple clones (1–9), mean MOI (3.1), and a lower number of distinct clones (21) compared to our findings in anophelines (Das et al. unpublished). The initial human MOI results are consistent with MOIs reported across Africa [13, 16, 26, 43, 63–66], and therefore suggests that there may be clones that are undetectable in humans, but equally transmitted and potentially more abundant in mosquitoes. More extensive studies that coordinate both mosquito and human blood specimen collections longitudinally will be required to evaluate inoculation rates of genetically diverse parasite clones by mosquitoes, vector biting heterogeneities, and the subsequent effect in the human population. Additionally, there have been reports of both *P. falciparum* and *P. ovale* co-infections in Nchelenge District (personal communication, Mbanga Muleba). The detection and influence of mixed infections of non-conspecific strains, if any, on *P. falciparum* MOI would certainly add to the body of knowledge regarding the complex interaction of multiple infections, clone development in both mosquito vectors and humans, and possible clinical outcomes.

The observed MOI in the mosquito abdomen, which was used for genotyping in this study, may be overestimated due meiotic recombination giving rise to numerous distinct clones, of which some may be selected against during ookinete crossing of the midgut. Accordingly, the lower MOI in the sporozoite stage supports the similarity in MOI between gametocytes in the human host and sporozoites in the anopheline vector as described by Morlais et al. [15]. However, Morlais et al. also reported that there were some oocyst and sporozoite allelic polymorphisms that were not detected in gametocytes, and this was attributed to imperfect detectability of minor clones in asymptomatic carriers and mosquitoes [15]. Moreover, when comparing vector and human MOI, a study in the Gambia found multiple oocyst clones in infected mosquitoes that were undetected in infected human blood samples, and the rate of distinct parasite alleles in the vector was much higher than would be expected by *P. falciparum* meiotic recombination patterns [13, 15]. Thus, these parasite clones may exist below the limit of detection in the human host, but thrive inside the mosquito where genetic diversity is maintained or expanded and novel clones are produced [13]. Together, the modeling and Gambia studies suggest that the parasite MOI observed in the mosquito midgut most likely reflects the clones that can evade both the human and mosquito immune systems and circulate within an endemic area such as Nchelenge. Future studies comparing the MOI of malaria oocyst and sporozoite stages in mosquitoes and their relationships with mono- or multiclonal gametocytes in both the vector and human hosts should be performed to better understand parasite transmission dynamics.

There were three main limitations in this study. First, for detection of multiple blood meals in anophelines, the method used to identify different hosts in a blooded mosquito was based on the three-allele method previously described by Norris et al. [4]. Previous studies in Kenya and Tanzania have used 6-locus and 8-locus fingerprints because several loci increase detection of numerous alleles that can be matched to fingerprints of clinical human blood specimens [40, 46]. The simulation model from which the three-allele method is based upon, indicated that a missed detection rate of 30–32% results in only a 3–5% bias [4]. In this study, the missed detection rate was smaller, 27.5%, suggesting a bias lower than that predicted by the Norris et al. model. It should be noted that a large proportion of the failed samples were from anophelines that were visually scored as unfed but found to be blooded by PCR; genotyping failure was likely due to low human DNA concentration in these mosquitoes. For the purposes of this study, the three-allele method was likely sufficient to identify the presence or absence of multiple blood meals, although a future study that includes more loci and matches mosquito blood meals to human genetic fingerprints would be informative. Secondly, for the MOI studies, the number of distinct alleles may have been underestimated by choosing the conservative 40 bp bin width used in this study to discern bands on multiple gels with varying electrophoretic migration of DNA fragments [44]. A more sensitive method like microsatellite analysis would help quantify the bias in PCR-based allele counts. Finally, the targeted PCR method used in this study for *P. falciparum* clones is not the most accurate method of measuring MOI; recently, microsatellite analysis has shown increased sensitivity in clone detection [15]. However, this method is expensive, requires special equipment and personnel training, and is not practical for field use. In this study, it was observed that the *glurp* locus was a poor marker for MOI detection by PCR, which by itself would have provided limited information. The addition of two more loci, *msp-1* and *msp-2*, was critical in identifying *P. falciparum* clones, indicating that when multiple loci are included, PCR can be a sufficient technique for measuring MOI. Accordingly, PCR can provide an adequate estimation of clonal parasite populations to observe epidemiological changes in field settings. However, if possible, microsatellite studies and other novel genetic or genomic methods should be used to complement PCR methods for more in-depth characterization of parasite clones in Nchelenge.

## Conclusions

Although multiple blood feeding behavior of mosquitoes and parasite multiplicity of infection are not part of the traditional malaria EIR measurement, molecular assessment of these parameters reveals additional complexity, which in this case increased existing EIR estimates. The investigation of MBF and MOI also provides additional insight into vector foraging behavior and parasite genetic diversity in a high transmission setting. The anopheline MBF rates and *P. falciparum* MOI in Nchelenge during the 2013 wet season were some of the highest recorded compared to that of humans in Africa. MBF behavior may also sustain already circulating clones and contribute to the generation of distinct parasites in the mosquito through repeated and potentially heterogeneous feedings on multiple individuals. Future studies should further define these parameters to inform how foraging behavior may influence the generation and evolution of parasites in holoendemic settings. These data suggest that anopheline MBF and MOI studies may serve as helpful tools in surveillance and research programs; the detection of changes in feeding behavior and malaria parasite genetic diversity may identify heterogeneities in malaria risk and changes in parasite population structure that will contribute to our understanding and development of effective malaria control strategies.

## Abbreviations

ACT: Artemisinin-based combination therapy
CDC LT: Center for Disease Control light trap
CSP: Circumsporozoite protein
*Cytb*: Cytochrome *b*
DBS: Dried blood spot
EIR: Entomological inoculation rate
ELISA: Enzyme-linked immunosorbent assay
*Glurp*: Glutamate receptor protein
HBR: Human biting rate
ICEMR: International Centers of Excellence in Malaria Research
IRS: Indoor residual spray
LLIN: Long lasting insecticide-treated net
MBF: Multiple blood feeding
MOI: Multiplicity of infection
*msp-1*: Merozoite surface protein 1
*msp-2*: Merozoite surface protein 2
PCR: Polymerase chain reaction
PSC: Pyrethroid spray catch
R_o_: Peproductive number
RDT: Rapid diagnostic test
RFLP: Restriction fragment length polymorphism
SD: Standard deviation
SIR: Sporozoite infection rate
